# *Sophora* genomes provide insight into the evolution of alkaloid metabolites along with small-scale gene duplication

**DOI:** 10.1186/s12864-023-09516-w

**Published:** 2023-08-22

**Authors:** Yang Jae Kang, Halim Park, Yejin Lee, Sanghwa Yoon, Myounghai Kwak

**Affiliations:** 1 Division of Bio & Medical Bigdata Department (BK4 Program), Gyeongsang National University, Jinju, 52828 Republic of Korea; 2 Division of Life Science Department, Gyeongsang National University, Jinju, Republic of Korea; 3 National Institute of Biological Resources, Incheon, 22689 Republic of Korea

**Keywords:** Congeneric, Divergence, Evolution, Functional annotation, Genome, Phylogeny, Secondary metabolite, Small-scale duplication, *Sophora*

## Abstract

The genus *Sophora* (Fabaceae) includes medicinal plants that have been used in East Asian countries since antiquity. *Sophora flavescens* is a perennial herb indigenous to China, India, Japan, Korea, and Russia. Its dried roots have antioxidant, anti-inflammatory, antibacterial, apoptosis-modulating, and antitumor efficacy. The congeneric *S. koreensis* is endemic to Korea and its genome is less than half the size of that of *S. flavescens*. Nevertheless, this discrepancy can be used to assemble and validate the *S. flavescens* genome. A comparative genomic study of the two genomes can disclose the recent evolutionary divergence of the polymorphic phenotypic profiles of these species. Here, we used the PacBio sequencing platform to sequence and assemble the *S. koreensis* and *S. flavescens* genomes. We inferred that it was mainly small-scale duplication that occurred in *S. flavescens*. A KEGG analysis revealed pathways that might regulate the pharmacologically important secondary metabolites in *S. flavescens* and *S. koreensis*. The genome assemblies of *Sophora* spp. could be used in comparative genomics and data mining for various plant natural products.

**Supplementary Information:**

The online version contains supplementary material available at 10.1186/s12864-023-09516-w.

## Introduction

*Sophora* spp. (Fabaceae) include medicinal plants that have been traditionally used since antiquity in East Asian countries. *Sophora* plants are sedative, depressant, analgesic, hypothermic, antitumor, antipyretic, and cardiotonic [1]. *Sophora* alkaloids were considered the major active chemical constituents. Researchers have already endeavored to isolate and identify these compounds [2]. The genus *Sophora* includes ~ 70 species that are widely distributed in tropical and temperate regions [3].

*Sophora flavescens* is a perennial herb native to China, India, Japan, Korea, and Russia. It is a well-known traditional medicine that has been extensively referenced in ancient writings. The common name for *S. flavescens* is “고삼(苦蔘) [gosam]” which translates as “bitter-tasting ginseng”. This name might reflect the presence of high concentrations of secondary metabolites in the plant. *Sophora flavescens* is still widely used as an herb and its dried roots have antioxidant, anti-inflammatory, antibacterial, apoptosis-modulating, and antitumor activity [4–8]. Its primary active ingredients and commercial quality indicators are matrine and oxymatrine. The former may have therapeutic efficacy against several diseases as it inhibits the inflammatory response and apoptosis [9].

*Sophora koreensis* is congeneric to *S. flavescens* and might also have ethnopharmacological efficacy. However, relatively less is known about this species as it is rare and legally protected in South Korea. While *S. flavescens* exhibits erect rhizomes, leaves measuring 15–25 cm in length, and leaflets measuring 2–4 cm with an obtuse to acute apex, *S. koreensis* displays creeping rhizomes, leaves measuring 4–6 cm in length, and leaflets measuring 0.8-1 cm with a rounded or emarginate apex. Both species are commonly found in grassy areas and thickets, but *S. koreensis* is strictly limited to the northern part of the Korean peninsula. In the roots of *S. koreensis*, only five compounds, namely Echinoisoflavanone, Kenusanone A, Echinoisosophoranone, Maackiain, and Medicarpin, have been identified. However, a total of 61 compounds have been identified from the roots of *S. flavescens* [10]. The genome of *S. koreensis* is less than half the size of that of *S. flavescens*. We hypothesized that by comparing these two genomes, we could facilitate the assembly and validation of that for *S. flavescens* and disclose the recent evolutionary divergence between these species in terms of their polymorphic phenotypic profiles. As *S. koreensis* has the smaller genome, it can be readily profiled for genome sequencing and gene cataloguing. Closely related species that widely differ in genome size may help explain the rapid evolution of the genes and genomes revealed by their gene catalogs and potentially regulating their medicinally relevant secondary metabolites.

We used the PacBio platform to sequence and assemble the *S. koreensis* and *S. flavescens* genomes. The former confirmed the expected genome size, and nearly all possible genes were predicted. The genome and gene catalog were profiled using various bioinformatics pipelines. The expected genome size was obtained from the *S. flavescens* genome assembly, and a gene catalog was prepared. Based on the number of duplicated gene pairs and their potential duplication modes, we inferred that small-scale duplication contributed to the expansion of the *S. flavescens* genome. We also observed that the transporter gene families differentially evolved between the species. A KEGG analysis revealed pathways that may regulate the pharmacologically important metabolites in *S. flavescens* and *S. koreensis*. The genome assembly of *Sophora* spp. could also be applied towards comparative genomics and data mining for future drug development.

## Materials and methods

### Plant materials

*Sophora flavescens* and *S. koreensis* were collected from their natural habitats in Gimpo, South Korea and the conservatory of the National Institute of Biological Resources (NIBR), respectively. Their voucher specimens were deposited at the NIBR herbarium (KE, *S. flavescens* NIBRVP0000729352; *S. koreensis* NIBRVP0000729369 identified by Myounghai Kwak). Their leaves, stems, roots, flowers, and fruits were frozen for RNA-Seq analysis and their fresh leaves were used in whole-genome sequencing (WGS). Plant collection methods were performed in accordance with the relevant guidelines and regulations.

### NGS sequencing

#### Sequel sequencing and library construction

The Covaris G-tube (Covaris, LLC, Woburn, MA, USA) was used to cut 20-kb genomic DNA segments according to the manufacturer’s protocol. An AMpureXP Bead Purification System (Beckman Coulter Life Sciences, Brea, CA, USA) was used to remove small fragments. Each sample weighed 5 g and was used to create the sequencing library. The SMRTbell® Express Template Preparation Kit (No. 101-357-000; Pacific Biosciences, San Diego, CA, USA) was used to create the SMRTbell library. SMRT cells and Sequel Sequencing Kit v. 3.0 (Pacific Biosciences) were used to sequence the SMRTbell library. The PacBio Sequel Sequencing Platform (Pacific Biosciences) was used to perform real-time sequencing on each SMRT Cell 1 M v. 3, and a 10-h run time was required per unit.

### WGS library construction and Illumina sequencing

1% agarose gel electrophoresis and a Qubit dsDNA HS Assay Kit (Thermo Fisher Scientific, Waltham, MA, USA) were used to verify DNA accuracy. The DNA library was constructed using a Truseq Nano DNA Library Kit (Illumina, San Diego, CA, USA) and a Nextera Mate Pair Library Prep Kit (Illumina) according to the manufacturer’s instructions. To produce DNA fragments of the desired size, 0.2 µg high-MW genomic DNA was randomly selected for sample library preparation in the Covaris S2 System (Covaris, LLC). Capillary electrophoresis and a Bioanalyzer (Agilent Technologies, Santa Clara, CA, USA) were used to assess the reliability of the amplified libraries. For RNA-Seq, total RNA was extracted from the flowers, buds, berries, roots, and stems of *S. koreensis* and the flowers, leaves, roots, and stems of *S. flavescens*. The RNA libraries were sequenced on a NovaSeq 6000 Platform (Illumina).

### Genome assembly

NextDenovo (https://github.com/Nextomics/NextDenovo) and Falcon (https://github.com/PacificBiosciences/FALCON) were used to assemble the PacBio long reads into contigs [11]. QuickMerge (https://github.com/mahulchak/quickmerge) was used to combine the assembled contigs into a single set [12]. The short reads generated by Illumina were used to correct the assemblies in NextPolish (https://github.com/Nextomics/NextPolish). PurgeHaplotig (https://github.com/skingan/purge_haplotigs_multiBAM) was used to remove redundant assemblies that could be allelic variants [13].

### Genome annotation

#### Repeat masking

RepeatModeler (http://www.repeatmasker.org/RepeatModeler/) was run in the RMBlast Engine (http://www.repeatmasker.org/RMBlast.html) for *de novo* repeat element mining. The LTR_Struct in the repeat modeling pipeline was used for long terminal repeat (LTR) mining. RepeatModeler and LTR Struct constructed repeat libraries for *S. flavescens* and *S. koreensis*. RepeatMasker (repeatmasker.org) was used to annotate the repeat regions on the genome.

### Gene prediction

After the annotated repetitive regions in the genome were masked, the genes were identified by Augustus-based gene prediction. Each species-specific gene prediction parameter originated from the BUSCO “viridiplantae_odb10” conserved gene set (https://busco-archive.ezlab.org/v3/frame_plants.html). Genes in the repeat-masked genome sequences were predicted using Augustus (http://bioinf.uni-greifswald.de/augustus/). The gene model was updated by aligning the RNA-Seq and genome sequences. HISAT2 (http://daehwankimlab.github.io/hisat2/) was used to align the Illumina RNA-Seq data. StringTie (https://ccb.jhu.edu/software/stringtie/) was used to reconstruct the transcripts from the alignments. Relative to the Augustus-based gene/transcript boundary prediction, the transcript assemblies reconstructed from RNA-Seq alignments were updated to include additional isoforms. GenomeTools (genometools.org) was used to verify the integrity of the gff3 annotation file [14]. The gene annotation files for *S. flavescens* and *S. koreensis* used in the present study are listed in Supplementary Files 1 and 2, respectively.

### Phylogenetic analysis

The core genes were selected for the nuclear genome-based phylogenetic tree based on their protein alignments and degrees of conservation. The eggNOG5 database (http://eggnog5.embl.de/#/app/home) was parsed into gene counts for each gene family to select the core gene families. For all land plants, conserved gene families must contain ≥ 1 and < 4 genes. The selected core gene families were represented with eggNOD I.D. Nos. 37HWA, 37JXH, 37MUB, 37QJC, and 37QRJ. The protein alignments were applied towards the Bayesian tree construction pipeline in BEAST (beast.community) [15]. The best-fit model of protein alignment evolution for the selected gene families was “Jones-Taylor-Thornton (JTT)” and it was estimated with ModelTest-NG (https://github.com/ddarriba/modeltest) [16].

### Gene family evolution analysis

Gene family evolution was analyzed with Phylip-dollop (https://github.com/felsenst/phylip/blob/master/dollop.c) [17] and by machine learning [18]. The land plant species annotations in the eggNOG database and the *Sophora* annotations from the present study (Supplementary File 3) were used to build the dataset. The default software settings were used in the Phylip-dollop analysis. However, the species tree used was that which was constructed with BEAST. The random forest algorithm was selected for the machine learning strategy as it efficiently selects features explaining the labels provided. The gene copy duplication modes were determined and listed by using the MCscanX pipeline (https://github-wiki-see.page/m/DR-genomics/Genomics-pipelines/wiki/MCScanx) for synteny, tandem, and ectopic duplications [19] (Supplementary File 4).

## Results

### Genome assembly of *S. koreensis* and *S. flavescens*

The genomes of *S. koreensis* and *S. flavescens* were predicted to be 671 Mb and 1,799 Mb, respectively, by K-mer analysis [20] and GenomeScope (http://qb.cshl.edu/genomescope/) [21]. We assembled the *S. koreensis* and *S. flavescens* genomes and compared them against those of previously published Viridiplantae species.

The genomic sequences of *S. koreensis* and *S. flavescens* were generated by the PacBio and Illumina sequencing platforms (Supplementary Table 1). The PacBio sequencing platform generated 61 Gb and 126 Gb for *S. koreensis* and *S. flavescens*, respectively, and covered > 70-fold of the predicted genome sizes. Short reads (37 Gb and 57 Gb) were also generated for base correction of *S. koreensis* and *S. flavescens*, respectively.

**Fig. 1:**
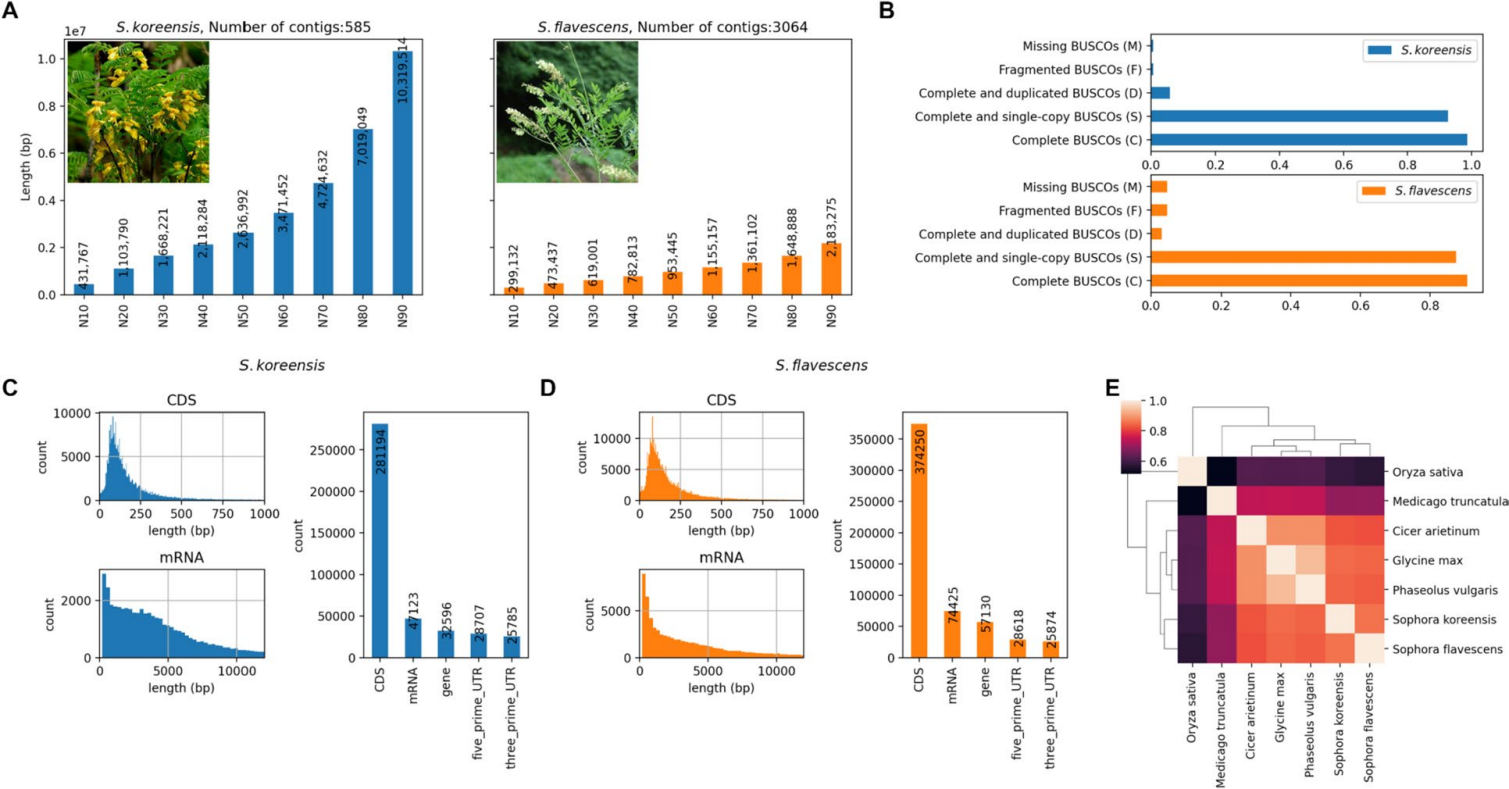
Genome assembly and prediction profiles of *S. koreensis and S. flavescens*. **A** Genome assembly evaluation based on N50 statistics. **B** Evaluations of completeness of genome assembly based on BUSCO. **C** Profile of predicted *S. koreensis* gene catalog. **D** Profile of predicted *S. flavescens* gene catalog. **E** Copy number correlation based on eggNOG orthologs among neighboring species

The *S. koreensis* and *S. flavescens* genomes were assembled using PacBio sequencing reads and NextDenovo (https://github.com/Nextomics/NextDenovo) and Falcon (https://github.com/PacificBiosciences/FALCON) assembly software. NextDenovo outperformed Falcon in terms of N50 values. However, the total number of NextDenovo assemblies revealed a smaller than expected genome size whereas the total number of Falcon assemblies met the predicted genome size. Therefore, we merged the Falcon and NextDenovo assemblies with Quickmerge (https://github.com/mahulchak/quickmerge) [12]. We also used PurgeHaplotigs (https://github.com/skingan/purge_haplotigs_multiBAM) to correct any potential duplications in the assemblies caused by allelic variants [13]. The resulting assemblies showed high contiguity and sufficed to extract gene catalogs with > 90% completeness according to a BUSCO analysis (Fig. 1A and B).

The PacBio reads for *S. koreensis* were assembled into 585 scaffolds totaling 623.88 Mb. The latter was in agreement with the estimated genome size. The PacBio reads for *S. flavescens* were assembled into 3,064 scaffolds totaling 1,665.47 Mb. The latter was slightly smaller than the estimated genome size and N50 = 953,445 bp. As expected, the *S. flavescens* assembly was significantly larger than that of *S. koreensis*.

To ensure the accuracy of both genome assemblies, a re-validation process was conducted by remapping short reads onto the assembled genome. This step aimed to confirm if the reads were mapped correctly, adhering to the paired-end library expectation for genome mapping. In the case of *S. flavescens*, all reads were successfully mapped, with 91.22% of them properly aligned to the genome. Similarly, for *S. koreensis*, 98.98% reads were successfully mapped, and 95.02% of them exhibited proper alignment to the genome. These results indicate that the genome assemblies are reliable.

### Comparative genome analyses by genomic annotation

#### Repeat and gene annotation

The RepeatMasker pipeline (repeatmasker.org) examined the assembled genomes and identified repetitive elements for each species (Supplementary Fig. 1). The DNA transposons and retrotransposons occupied the genomes, and their profiles distinctly differed between *S. koreensis* and *S. flavescens*. The latter had higher retrotransposon region/total genome size ratios than *S. koreensis*. Expansion of the LTR elements in the genome might account for the relatively larger *S. flavescens* genome (Supplementary Table 2). Gypsy/DIRS1 occupied twice as much of the *S. flavescens* genome as that of *S. koreensis*. A previous study showed that the euchromatic area in the pepper genome was twice as large as that in the tomato genome mainly because of differential accumulation of Gypsy-like elements [22].

We used ab initio and evidence-based approaches to predict genes based on the repeat masked genome sequences and to retrieve the gene catalogs of the assembled genomes. We implemented RNA-Seq in a tissue-specific manner (Supplementary Table 1). To cover as many transcript variants as possible for both species, we generated 76.42 Gb RNA-Seq data that included the flowers, leaves, roots, and stems. Based on the ab initio gene prediction approach of Augustus, we used StringTie (https://ccb.jhu.edu/software/stringtie/) to add extra transcripts from the RNA-Seq data [23, 24]. We found 32,596 and 57,130 predicted genes for *S. koreensis* and *S. flavescens*, respectively. More genes were discovered in the genomes of *S. flavescens* than in those of *S. koreensis*. Therefore, LTR expansion and gene duplication might explain genome size expansion in *S. flavescens*. The length distributions of the genomic region of CDS and mRNA resembled those in the predicted gene catalogs of both species. Nevertheless, there were relatively more short genomic regions for the mRNAs in the *S. flavescens* genome (Fig. 1 C and D).

#### *Genome size evolution of* Sophora *spp.*

**Fig. 2:**
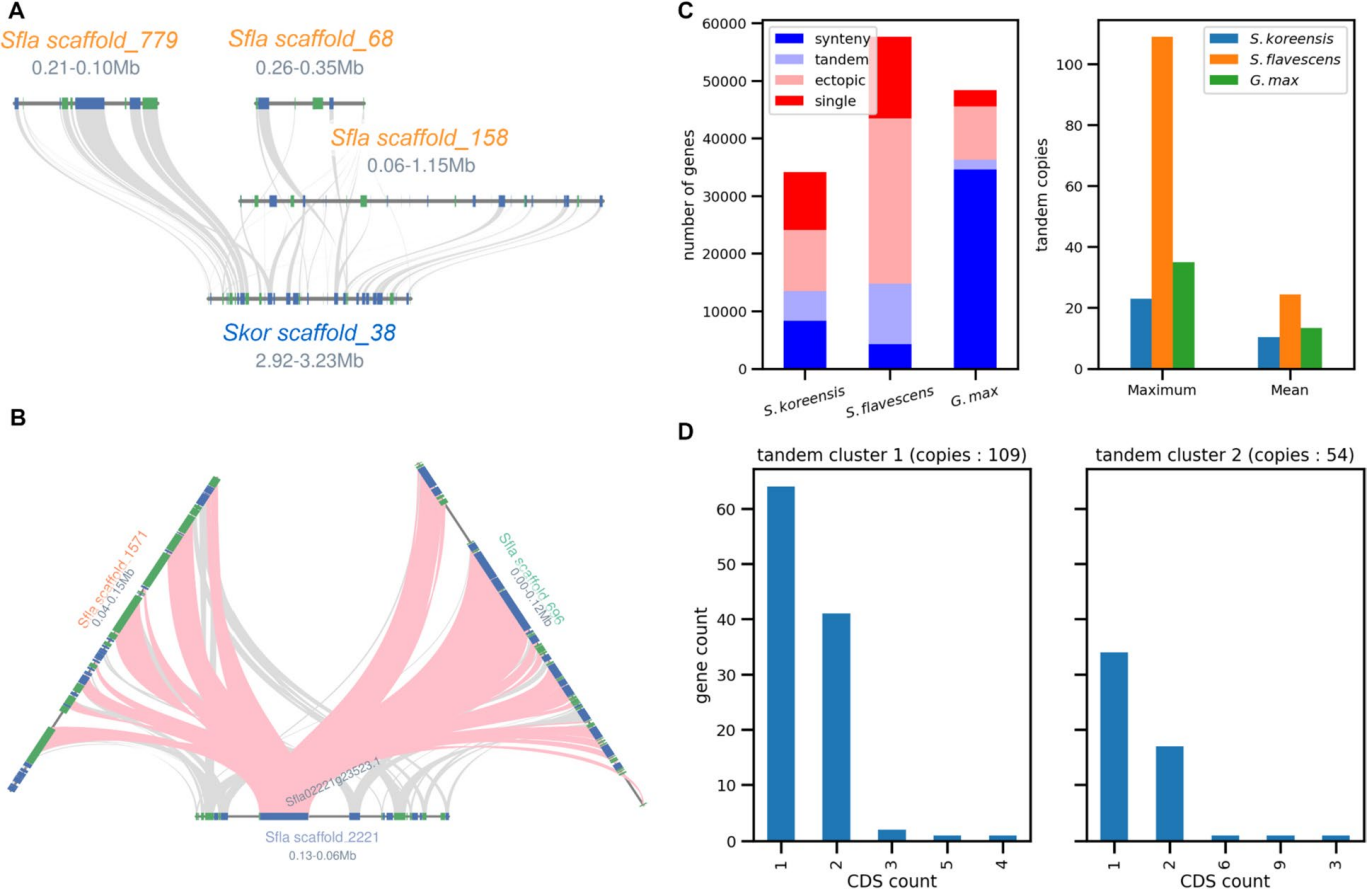
Interspecies gene collinearity conservation and possible duplication modes in genome size evolution from *S. koreensis and S. flavescens. ***A** Syntenic relationship between *S. koreensis* and *S. flavescens*. **B** Synteny block combined with extreme tandem duplications in *S. flavescens*. **C** Different gene duplication modes depicted by gene counts as well as maximum and mean copy numbers in tandem duplication blocks for each species. **D** CDS number distribution for tandem copies in two largest tandem clusters

We evaluated the *S. koreensis* and *S. flavescens* genomes by using their synteny relationships with *Glycine max.* The latter has been built into a nearly complete reference genome for the Fabales (Supplementary Fig. 2A**;** Fig. 2A). Close phylogenetic relationships between *Sophora* spp. and *Glycine* spp. could be inferred from the dense synteny block distribution. Ks values were calculated for the gene pairs along with the synteny blocks. The Ks histogram showed similar peaks for the comparisons between *G. max* and *S. flavescens* (Ks = 0.40) and between *G. max* and *S. koreensis* (Ks = 0.42). By contrast, the Ks histogram showed a lower peak (Ks = 0.12) for the comparison between *S. flavescens* and *S. koreensis*. These findings validated the speciation order. We also performed an intragenome synteny analysis for each species that enabled us to observe whole genome duplication (WGD) traces. We obtained Ks = 0.43 for *S. koreensis* (Supplementary Fig. 2B). The two *S. flavescens* peaks in the Ks histogram at 0.31 and 1.31 resembled those for *G. max* in a previous study. The latter demonstrate two WGD rounds based on the observed peaks in the Ks distribution [25]. There were significantly fewer synteny blocks on the peaks of *S. flavescens* than there were on those for *G. max* (Supplementary Fig. 2B). Hence, the WGD event may not be the major factor influencing the increases in the *S. flavescens* genome size.

#### *Small-scale duplication in* Sophora flavescens

We also investigated small-scale duplication traces revealed by individual gene homology. The *S. flavescens* genome showed intensive tandem duplication traces within the synteny blocks (Fig. 2B). The overall gene counts for the gene duplication modes disclosed dominant duplication events for each species (Supplementary File 4). There was large-scale and small-scale duplication for *G. max* and *S. flavescens*, respectively (Fig. 2C). Small-scale gene duplication frequently results in high copy numbers within particular gene families [26]. We discovered high copy numbers of tandemly duplicated gene families in the *S. flavescens* genome. The numbers of CDS copies in the largest tandem clusters indicated that nearly half the tandem copies had single CDS genes. Therefore, LTR activity duplicated the tandem copies (Fig. 2D). Retrocopies are made by RNA-based duplication. They are reverse-transcribed from mRNA without introns and then inserted into the genome at new locations [27].

#### Functional annotation of gene catalogs

The eggNOG-mapper software (eggnog-mapper.embl.de) implemented functional annotation and gene family classification to clarify their phenotypic impacts on gene catalog evolution [28, 29]. Shared copy number evolution within the Fabales was suggested based on the correlation of each species copy number profile. Thus, the gene catalogs predicted for *S. flavescens* and *S. koreensis* were reliable as they were clustered within the expected taxonomy (Fig. 1E).

#### Sophora koreensis *and* Sophora flavescens *speciation*.

**Fig. 3:**
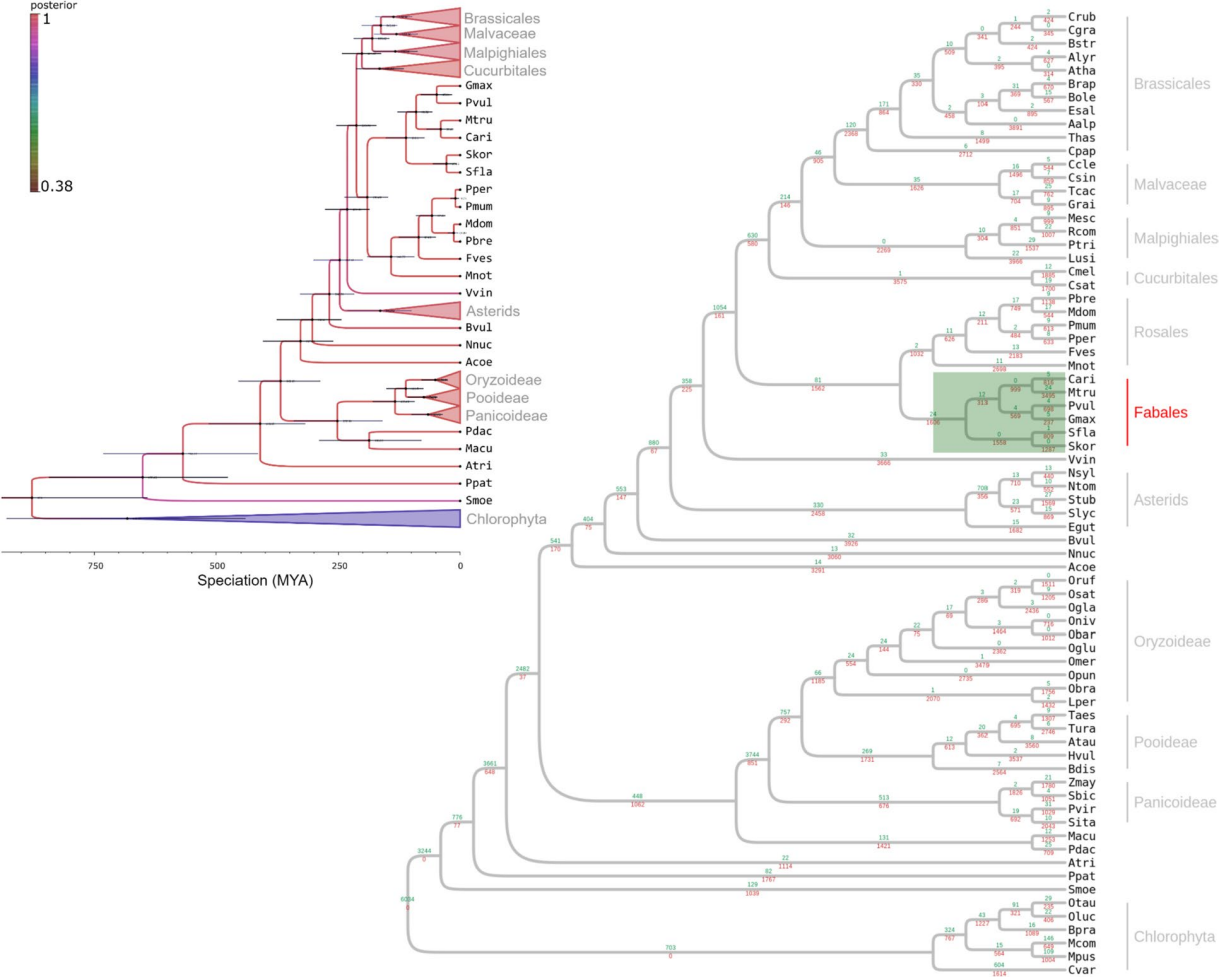
Bayesian speciation tree showing speciation times (left, top), gene family expansion (green #), and gene family contraction (red #) (right). Posterior probability of branch is indicated by branch color (colorbar)

We used Bayesian tree construction to elucidate *S. koreensis* and *S. flavescens* speciation based on genomic evidence. We selected five highly conserved gene families with comparatively few protein alignment gaps and minimal copy number variation among species. The protein alignments of the selected gene families were fed into the Beast pipeline (https://beast.community) [15]. We constructed a species tree using a total of 72 plants including 70 from the eggNOG database as well as *S. koreensis* and *S. flavescens* (Fig. 3). The species tree explained the phylogenetic relationships among the Fabales including *Cicer arietrium*, *Medicago truncatula*, *Phaseolus vulgaris*, *G. max*, *S. flavescens*, and *S. koreensis*.

We set the root divergence time between Phylum Chlorophyta and the land plants at 868 MYA (http://www.timetree.org/). We estimated the time of divergence between *G. max* and *P. vulgaris* as ~ 47.37 MYA. A recent phylogenetic study reported the divergence time for the same species pair as 43.8 MYA [30]. We estimated the speciation time for *S. koreensis* and *S. flavescens* to be ~ 27.6 MYA.

Based on a dollop analysis in Phylip (https://evolution.genetics.washington.edu/phylip.html), we listed the gained/lost gene families in the Fabales (Fig. 3; Supplementary Fig. 3). After species divergence, the Fabales clade lost many and gained few gene families. The gene families related to “signal transduction mechanisms” underwent most of the gain and loss events (Supplementary Fig. 3). For this reason, rewiring signal transduction might be the primary evolutionary factor in the Fabales.

**Fig. 4:**
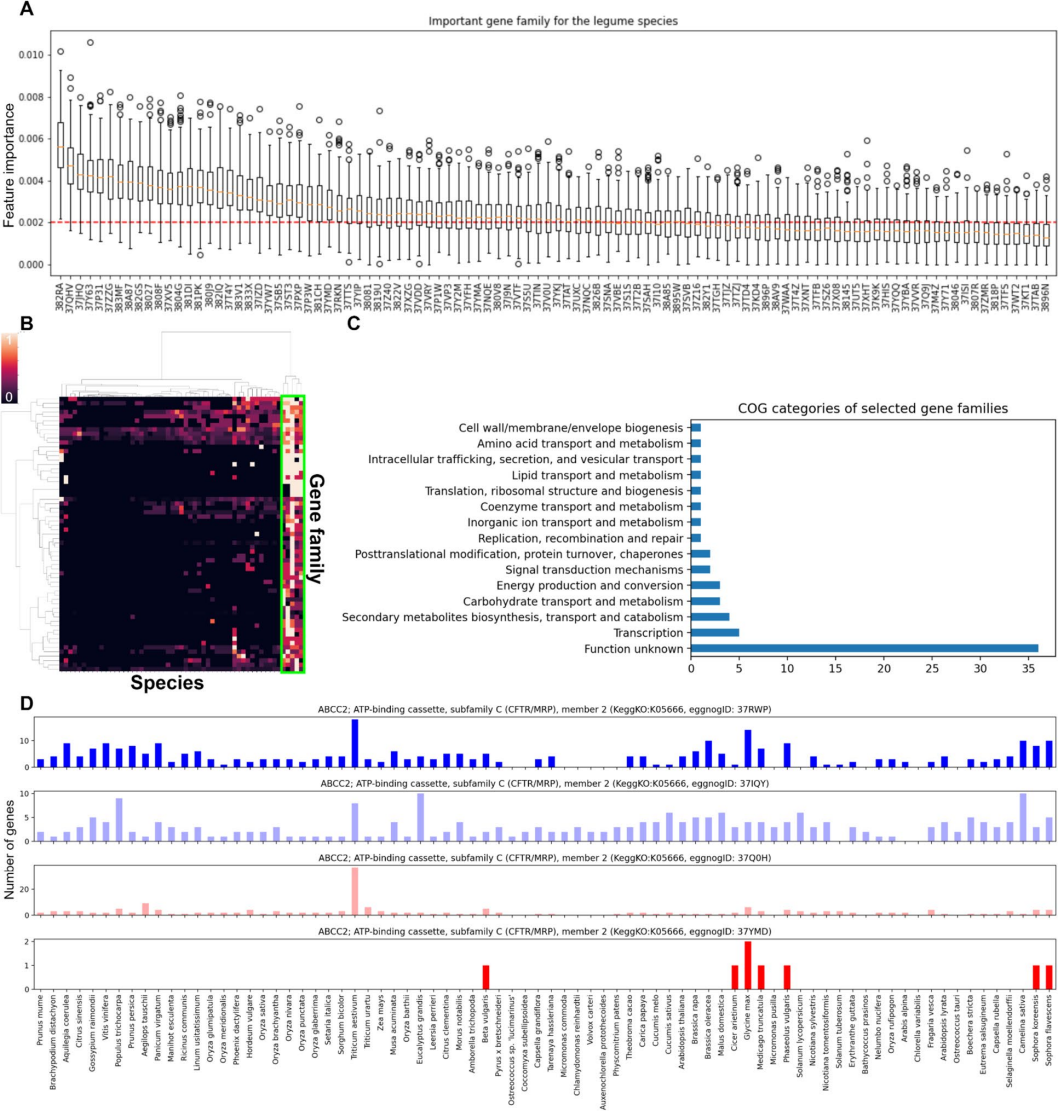
Comparative analysis of gene family counts based on eggNOG database and those of***S. koreensis***and***S. flavescens***. **A** Feature importance distribution from 200-fold iterative training of random forest algorithm classifying legume species *C. arietinum*, *G. max*, *P. vulgaris*, *S. koreensis*, *S. flavescens*, and others. Horizontal red line indicates feature importance threshold. **B** Heatmap showing normalized copy numbers of selected gene families by feature importance. **C** COG categories of selected gene families. **D** Copy number profile of ABCC gene families expressed in bar plot

The Phylip-dollop algorithm uses binary coding (presence or absence) of gene families. Thus, it may ignore quantitative increases in gene families that could be vital to environmental adaptation according to the tandem gene duplication analysis. We used machine learning for the quantitative identification of Fabales gene family growth. Highly expanded Fabales gene families may be detected by using a feature selection technique based on random forests. We used the labels provided, namely, “categorical classes: legume species and others” and captured the significant features via the random forest classification algorithms. Thresholds for the mean feature importance values were acquired after 200 iterative random forest trainings and disclosed a list of significantly elevated Fabales gene families (Fig. 4A and B). Gene families related to “transcription” constituted the majority under a single category. In contrast, the term “transport and metabolism” occurred under numerous categories and accounted for most of the totals (Fig. 4C). The increased copy number under “transport and metabolism” may be associated with the nitrogen fixation capacity of many legume species [31].

We focused on exploring specific gene families identified through machine learning techniques. Among these families, we aimed to highlight the ATP-binding cassette (ABC) subfamily C, member 2 (ABCC2) transporter. This transporter falls under the COG classification of ‘Secondary metabolites biosynthesis, transport and catabolism’ (Fig. 4D) and is known to play a crucial role in the transportation of various plant secondary metabolites, including alkaloids, terpenoids, flavonoids, volatile organic compounds (VOCs), and apocarotenoids [32]. Except for *Beta vulgaris*, the 37YMD subfamily of the ABCC families most frequently occurs in legumes such as *Glycine max*, *Medicago truncatula*, *Cicer arietinum*, and *Phaseolus vulgaris*. Furthermore, we conducted a genome analysis on additional Lupinus plant, *Lupinus albus*, known for its production of quinolizidine alkaloids. Our investigation revealed that *L. albus* also shares the 37YMD subfamily of ABCC families with other legume species (Supplementary Fig. 4). The alphafold2 database (https://alphafold.com) [33] and analyses of the ABCC protein structures revealed that 37YMD had lost its channel-like structure and could behave differently from other subfamilies (Supplementary Fig. 5). Additionally, we have checked a vacuolar membrane-localized multidrug and toxic compound extrusion (MATE) transporter (https://www.uniprot.org/uniprotkb/Q9LYT3). Previous report showed a preferential transport of epicatechin 3’-O-glucoside by this transporter [34]. While the MATE transporter protein families are generally involved in the translocation of secondary metabolites across various plant species [35], it is important to note that the specificity to legume species is not universally observed in our research. Nonetheless, we identified that one particular MATE gene family shows a high degree of copy number increase for *S. flavescens* (Supplementary Fig. 6).

#### *Pathway evolution between* Sophora koreensis *and* Sophora flavescens

**Fig. 5:**
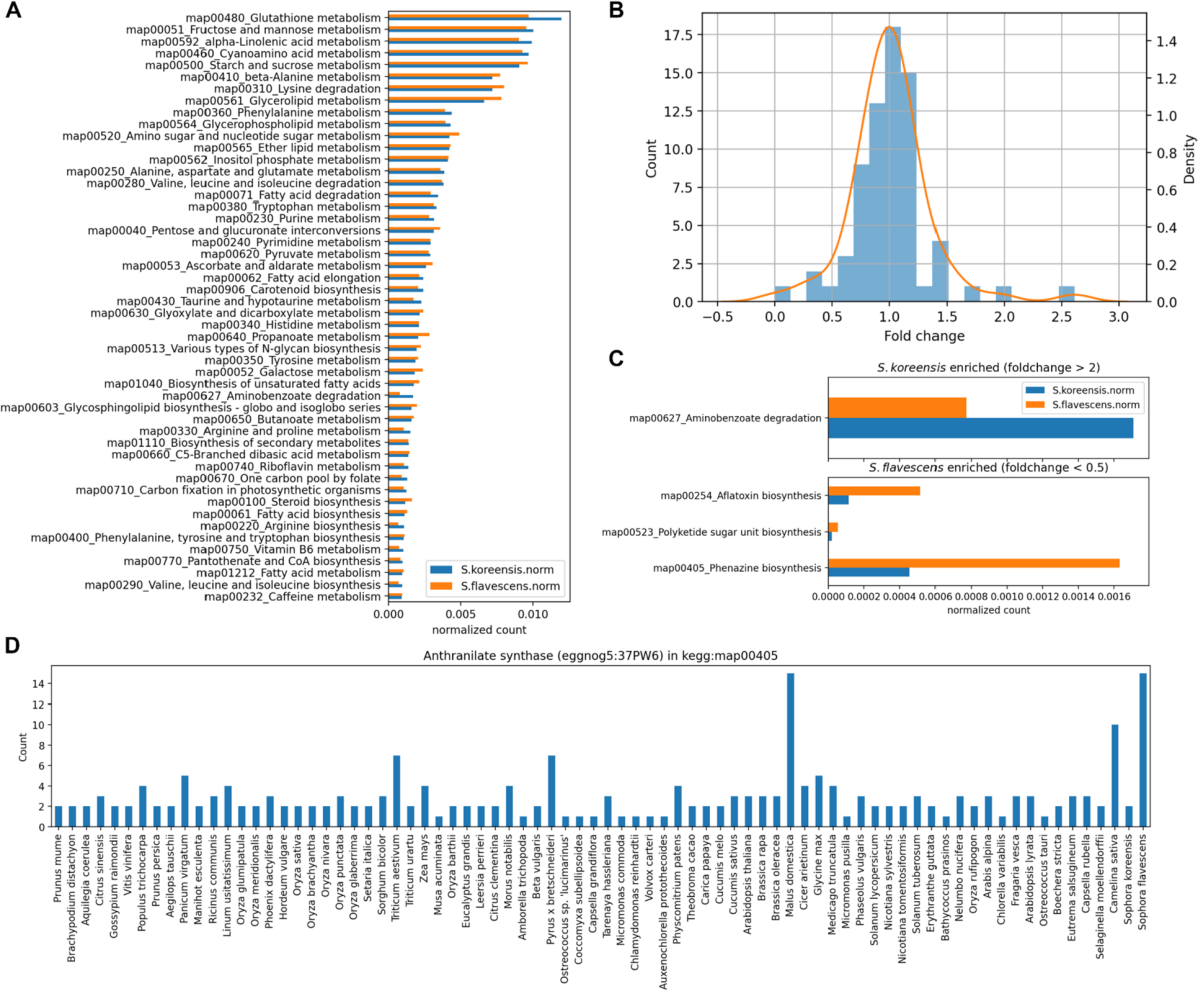
Gene count comparisons based on KEGG pathway classification.** A** Normalized gene counts for each KEGG pathway. **B** Fold change distributions of normalized gene counts in KEGG pathway between *S. koreensis* and *S. flavescens*. **C** Enriched pathways for each species based on twofold change difference. **D** Anthranilate synthase copy number distributions among land plants

Genomic information shared among *Sophora* species may disclose shared and distinct aspects of secondary metabolism and enable us to estimate metabolite profiles. We compared the gene catalogs of *S. koreensis* and *S. flavescens* with respect to the KEGG pathway to understand the unique evolution between species [36]. As *S. koreensis* and *S. flavescens* are phylogenetically close, there was overall consistency in the number of gene members in each KEGG pathway (Fig. 5A). Thus, the change in the number of gene members between congenerics was normally distributed (Fig. 5B). The phenazine biosynthesis (KEGG:map00405) pathway was highlighted when we used thresholds of 2 and 0.5 to retrieve gene count-affecting pathways between *S. koreensis* and *S. flavescens* (Fig. 5C). The number of genes in the phenazine biosynthesis pathway of *S. flavescens* was highly recorded because of the anthranilate synthase family (eggnog5:37PW6) (Fig. 5D). Anthranilate synthase catalyzes the conversion of chorismate to anthranilate. Prior research revealed that the medicinal plant *Catharanthus roseus* produces various terpenoid indole alkaloids and suggested that anthranilate synthase-catalyzed conversion from chorismate to anthranilate is the rate-limiting step in the indole pathway [37, 38]. The pharmacological and phytochemical activity of *S. flavescens* [39] may be linked to the fact that this species harbors relatively more anthranilate synthase family members (eggnog5:37PW6).

Moreover, we performed a comparative analysis between two species by utilizing the KEGG pathway “Tropane, piperidine and pyridine alkaloid biosynthesis” (map00960). The selection of this pathway was motivated by the well-known presence of matrine, a pivotal alkaloid compound, in *Sophora*. Matrine, along with oxymatrine, constitutes the primary active ingredients and serves as a crucial indicator of commercial quality. Through the pathway-based comparison (map00960), we observed an increased copy number of the amine oxidase gene family (37JD8) in *S. flavescens* compared to *S. koreensis* (Supplementary Fig. 7).

## Discussion

The estimated size of the *S. flavescens* genome is ~ 1.7 Gb. Thus, it is difficult to assemble it to extract sufficient genes. It is also challenging to confirm whether its predicted gene catalog is adequate for downstream analyses. The relatively small size of the cotton genome permitted the diploid species *Gossypium raimondii* to be sequenced and used as a reference for studying tetraploid cotton genomes [40]. Here, the combination of the *S. flavescens* genome and the assembly of the smaller congeneric *S. koreensis* enabled the validation of the predicted gene catalog of the former using comparative genomic approaches. Species clustering based on gene copy numbers showed that the predicted gene catalogs of *S. flavescens* and *S. koreensis* were localized to their expected places along with the Fabales (Fig. 1E). Therefore, the *S. flavescens* gene catalogs were accurate enough for use in the subsequent analyses.

After verifying the reliability of the assembled genome, we selected highly conserved gene families to estimate speciation time via a Bayesian tree. The *Sophora* clade was situated in an expected phylogenetic position. It was estimated that speciation between *S. flavescens* and *S. koreensis* occurred at 27.6 MYA. This age precedes any other known speciation times for *Glycine* spp. Previous research showed the maximum speciation time for *Glycine* spp. was 7.22 MYA [41]. The authors reported the phylogenomic results for nine *Glycine* species and created a draft genome for each of them. We consistently found that the speciation time in *Sophora* spp. was twice as early as the maximum speciation time in *Glycine* spp. even after normalization by adjusting the speciation time between *S. flavescens and S. koreensis* to 15 MYA based on the speciation time between *Glycine* spp. and *Phaseolus* spp. (Zhuang et al. [41],: 26 MYA; the present study: 47.37 MYA).

We attempted to clarify the evolution of the *S. flavescens* genome size based on *Sophora* genomes. We discovered a relative increase in the number of LTR repeats that seems to enlarge the *S. flavescens* genome. We used an intragenome synteny analysis based on the MCscanX pipeline [19] and found both ancient and modern duplication traces despite the weak signal. A self-BLAST analysis of the *S. flavescens* gene catalog produced a list of homologous genes indicating that single-gene duplication events lead to high gene family copy numbers. Tandem duplication in *S. flavescens* revealed a maximum of > 100 gene stretches. Based on the observations, small-scale rather than large-scale duplication may be the main cause (Fig. 2C). The increased number of small-scale duplication traces and the high LTR number in the *S. flavescens* genome may be related. A previous study suggested that flanking transposable elements caused the recent increase in the number of gene duplications in Triticeae species [42]. In the two largest tandem clusters in *S. flavescens*, > 50% of the tandem duplicates were intronless (Fig. 2D). As plants are sessile, it is difficult for them to adjust to changes in their ambient environment. Unlike animals, they cannot flee from danger. Hence, active tandem gene duplication and increases in copy number might enable plants to survive in the presence of various stressors (Kondrashov, 2012). These genetic modifications could enhance their resistance to a wide range of environmental threats. *Sophora flavescens* is known as “bitter-tasting ginseng”. It might have acquired this defensive trait by modulating its secondary metabolite composition in response to dramatic environmental changes that induced the alteration of its genome structure via LTR copy expansion.

Though *S. flavescens* and *S. koreensis* had different numbers of members in very few pathways, the manner in which their gene counts were distributed in the KEGG pathways was consistent across both species. The numerous genes predicted for *S. flavescens* were not annotated by a homology-based method, and the pathway analysis was not thorough enough to enable us to draw clear conclusions. Nevertheless, most known pathways were well conserved. Furthermore, the few pathways with different gene copy numbers would partially explain the variation in the metabolite profiles among *Sophora* spp. This phenomenon was exemplified by anthranilate synthase (Fig. 5D) [39].

Observation of the genes and genome evolution in the assemblies of *Sophora* spp. and mining of the model plant functions and pathway databases help elucidate the genomic basis of metabolite profiles. Our *Sophora* genome assemblies could help identify the impact of genomic factors on various secondary metabolites. They could also be used in pan-genome analysis of medicinal legume species and clarify the evolution of pharmacologically useful plant natural products.

### Supplementary Information

**Additional file 1: Supplementary file 1.** GFF file for *S. flavescens*. **Supplementary file 2.** GFF file for *S. koreensis*. **Supplementary file 3.** Gene family counts for land plants. *Sophora* species counts were calculated using the annotations by eggnog-mapper. **Supplementary file 4.** Classification of genes of *G. max*, *S. flavescence* and *S. koreensis* according to duplication types (synteny, tandem, or ectopic duplication).

**Additional file 2: Supplementary table 1.** Sequencing summary for *Sophora* species, *S. flavescens *and *S. koreensis*. **Supplementary table 2.**  Repeat profile of *Sophora* species and model plants; *G. max*, *A. thaliana* and *O. sativa*

**Additional file 3:** **Supplementary Figure 1.** Repeat content profiling of *S. flavescens* and *S.koreensis *genomes. **Supplementary Figure 2.** Ks distributions obtained by comparative legume-species analysis. **Supplementary Figure 3.** Legume species gene-family loss and gain based on Phylip-Dollop analysis. **Supplementary Figure 4.** Gene family counts of ABCC2, along with plant species, including additional legume species Lupinus plants known for their production of quinolizidine alkaloids. **Supplementary Figure 5.** 3D structure of the ABCC gene family predicted with alphafold2. **Supplementary Figure 6. **Copy number plot of MATE orthologs (KEGG ID: K03327) within the gene family profile. Notably, the 37MMA species shows a remarkable specificity for *S. flavescens*. **Supplementary Figure 7.** Increased copy number of amine oxidase (eggnog: 37JD8) in *S. flavescens *compared to *S. koreensis* based on Kegg pathway [36], map00960 (Tropane, piperidine and pyridine alkaloidbiosynthesis), highlighting differences in alkaloid synthesis between the two species. The red boxes indicate the amine oxidase in pathway and the bar plot of the copy numbers of gene families.

## Data Availability

The raw sequence reads were deposited in the SRA under BioProject No. PRJNA892681. The assembled *S. flavescens* and *S. koreensis* sequences of data are available on NCBI under BioSampleID Nos. SAMN31373329 and SAMN31373487.
