# Supplementary material for: Sophora genomes provide insight into the evolution of alkaloid metabolites along with small-scale gene duplication
Source: BMC Genomics. 2023 Aug 22;24:475. doi: 10.1186/s12864-023-09516-w (PMC10464357; doi:10.1186/s12864-023-09516-w)
Supplement: Supplementary file 3 — Additional file 3: Supplementary Figure 1. Repeat content profiling of S. flavescens and S.koreensis genomes. Supplementary Figure 2. Ks distributions obtained by comparative legume-species analysis. Supplementary Figure 3. Legume species gene-family loss and gain based on Phylip-Dollop analysis. Supplementary Figure 4. Gene family counts of ABCC2, along with plant species, including additional legume species Lupinus plants known for their production of quinolizidine alkaloids. Supplementary Figure 5. 3D structure of the ABCC gene family predicted with alphafold2. Supplementary Figure 6. Copy number plot of MATE orthologs (KEGG ID: K03327) within the gene family profile. Notably, the 37MMA species shows a remarkable specificity for S. flavescens. Supplementary Figure 7. Increased copy number of amine oxidase (eggnog: 37JD8) in S. flavescens compared to S. koreensis based on Kegg pathway [36], map00960 (Tropane, piperidine and pyridine alkaloidbiosynthesis), highlighting differences in alkaloid synthesis between the two species. The red boxes indicate the amine oxidase in pathway and the bar plot of the copy numbers of gene families. [file 12864_2023_9516_MOESM3_ESM.doc]

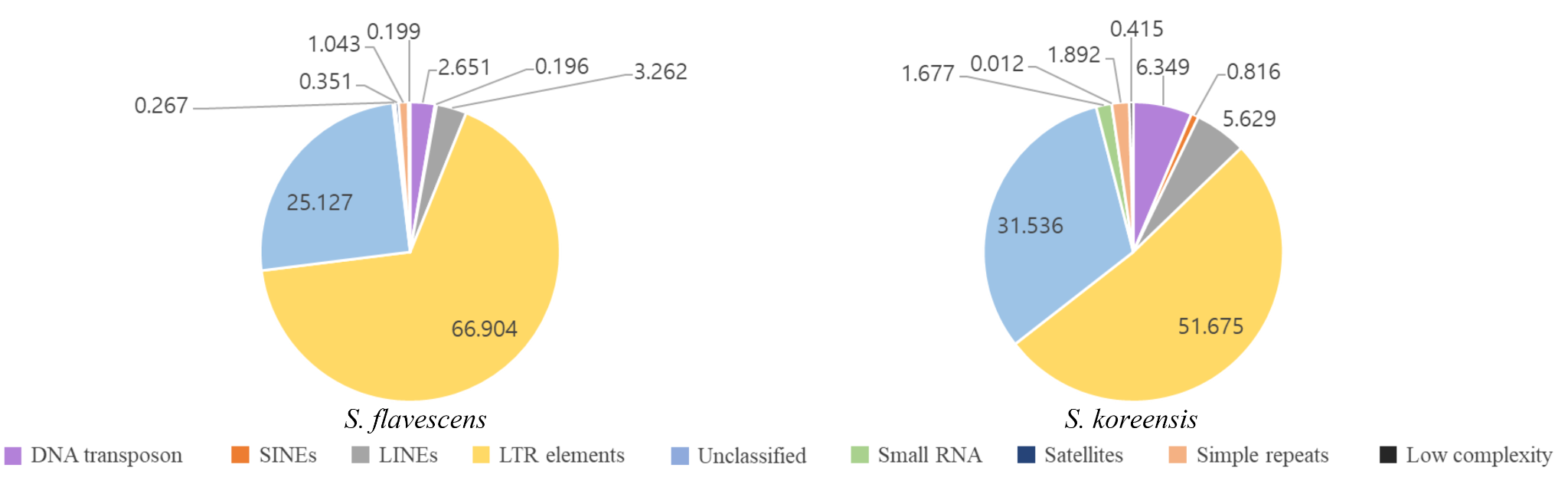


**Supplementary Figure 1. Repeat content profiling of *S. flavescens* and *S. koreensis* genomes.**


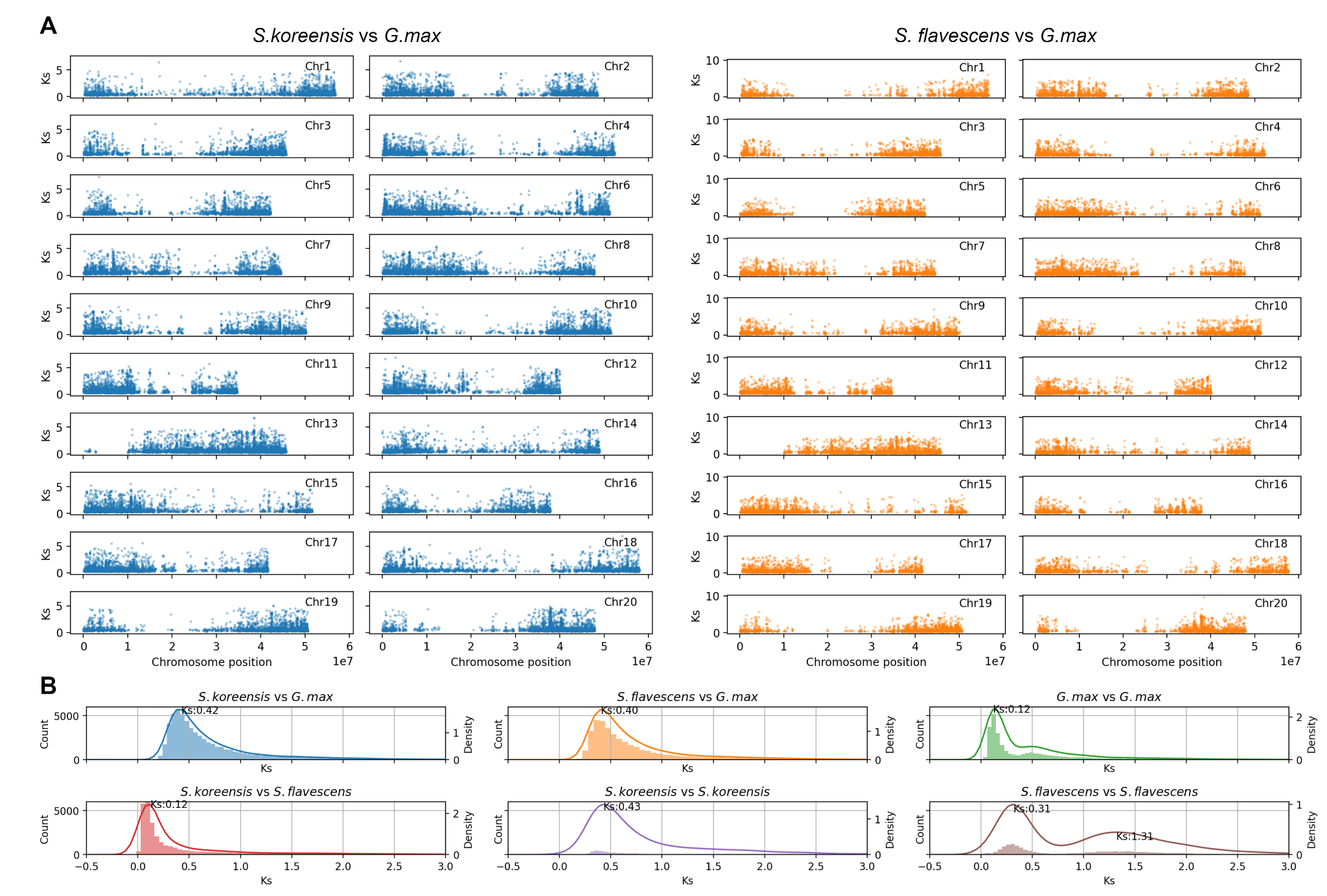


**Supplementary Figure 2. Ks distributions obtained by comparative legume-species analysis.**


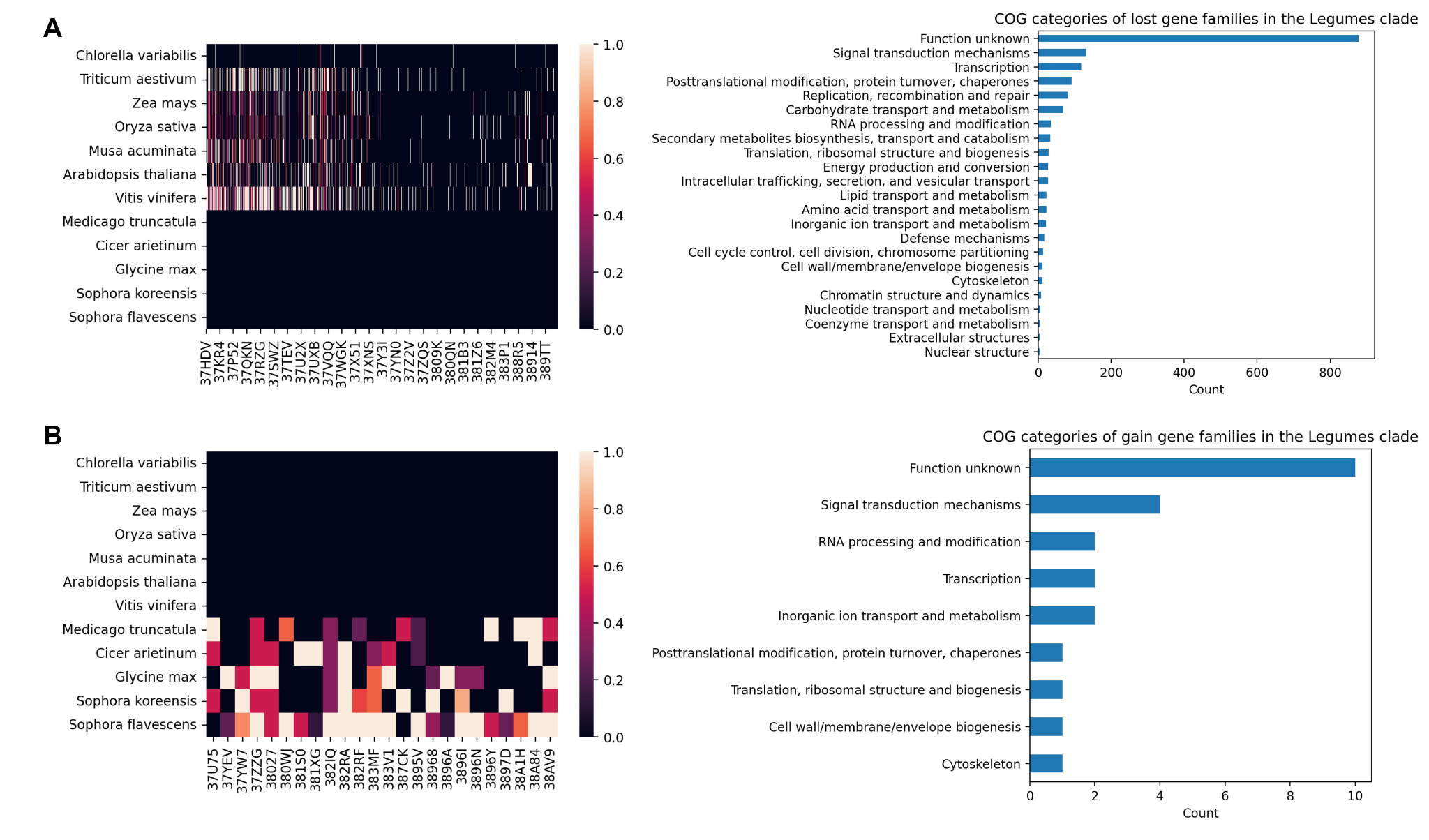


**Supplementary Figure 3. Legume species gene-family loss and gain based on Phylip-Dollop analysis.**

**
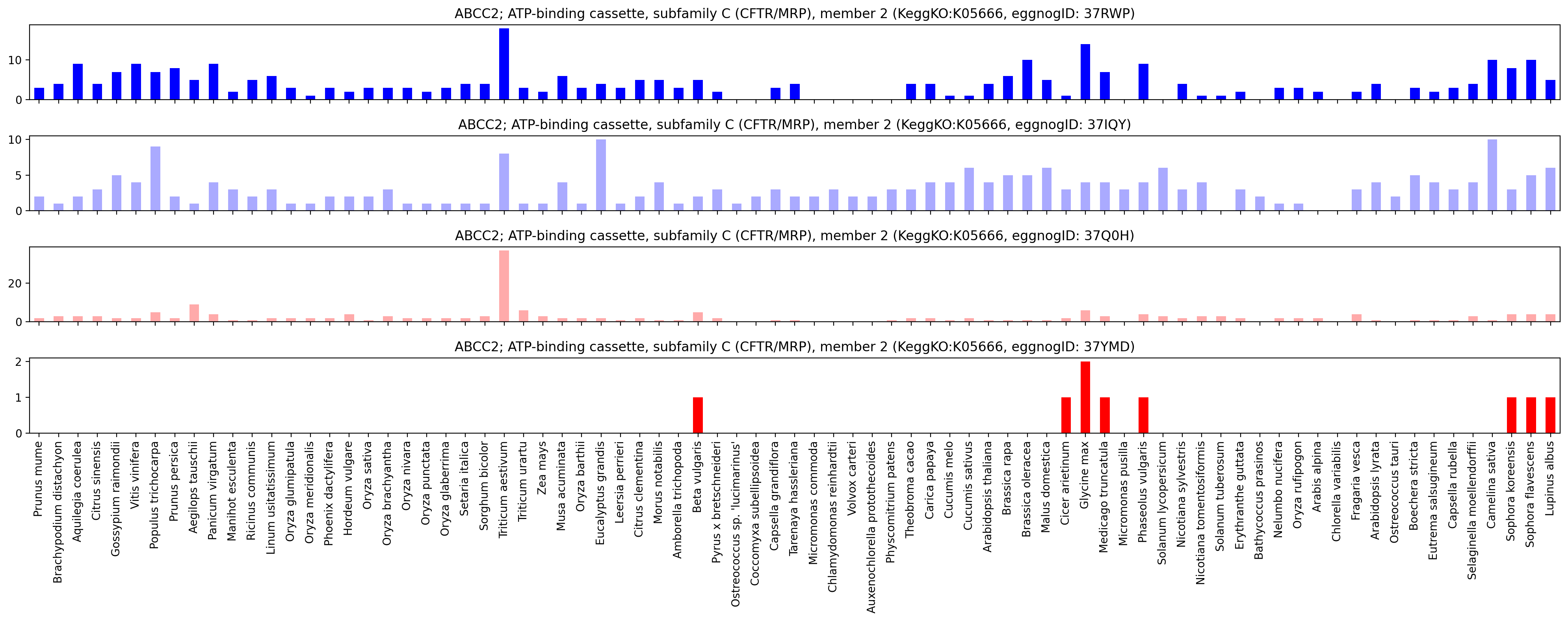
**

**Supplementary Figure 4.** Gene family counts of ABCC2, along with plant species, including additional legume species Lupinus plants known for their production of quinolizidine alkaloids.


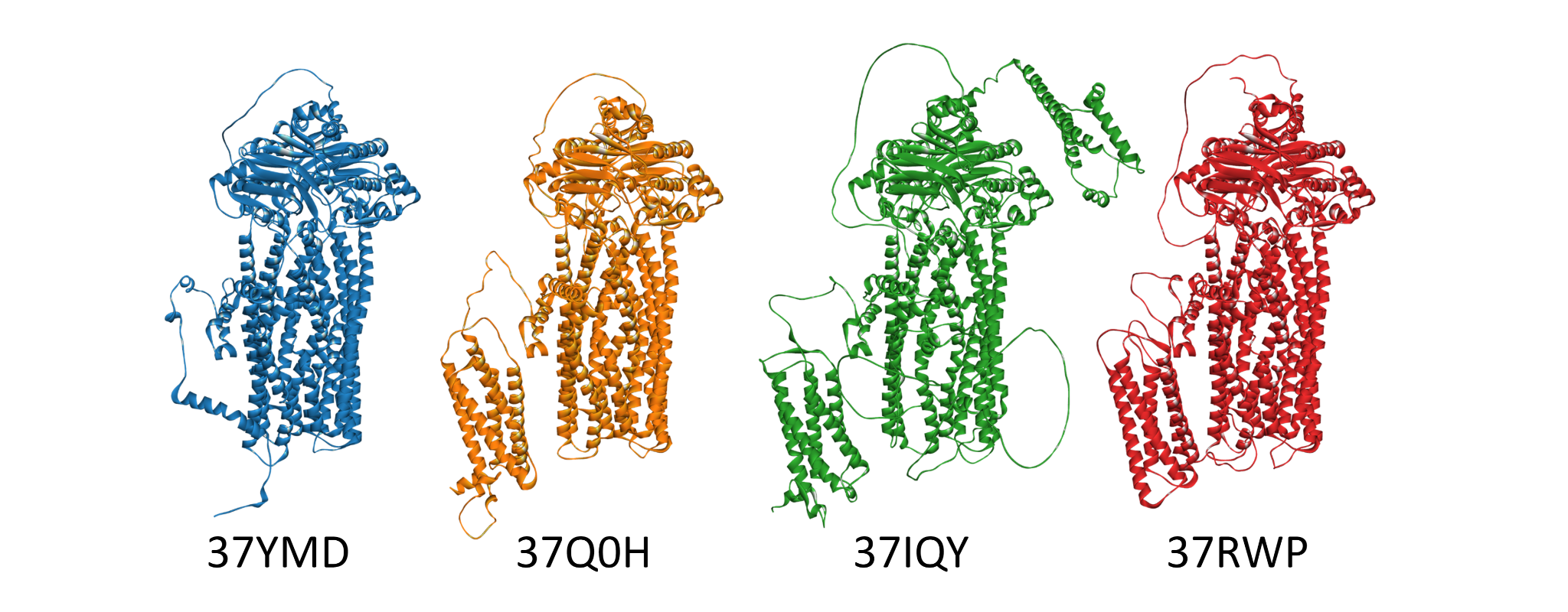


**Supplementary Figure 5. 3D structure of the ABCC gene family predicted with alphafold2.**

**
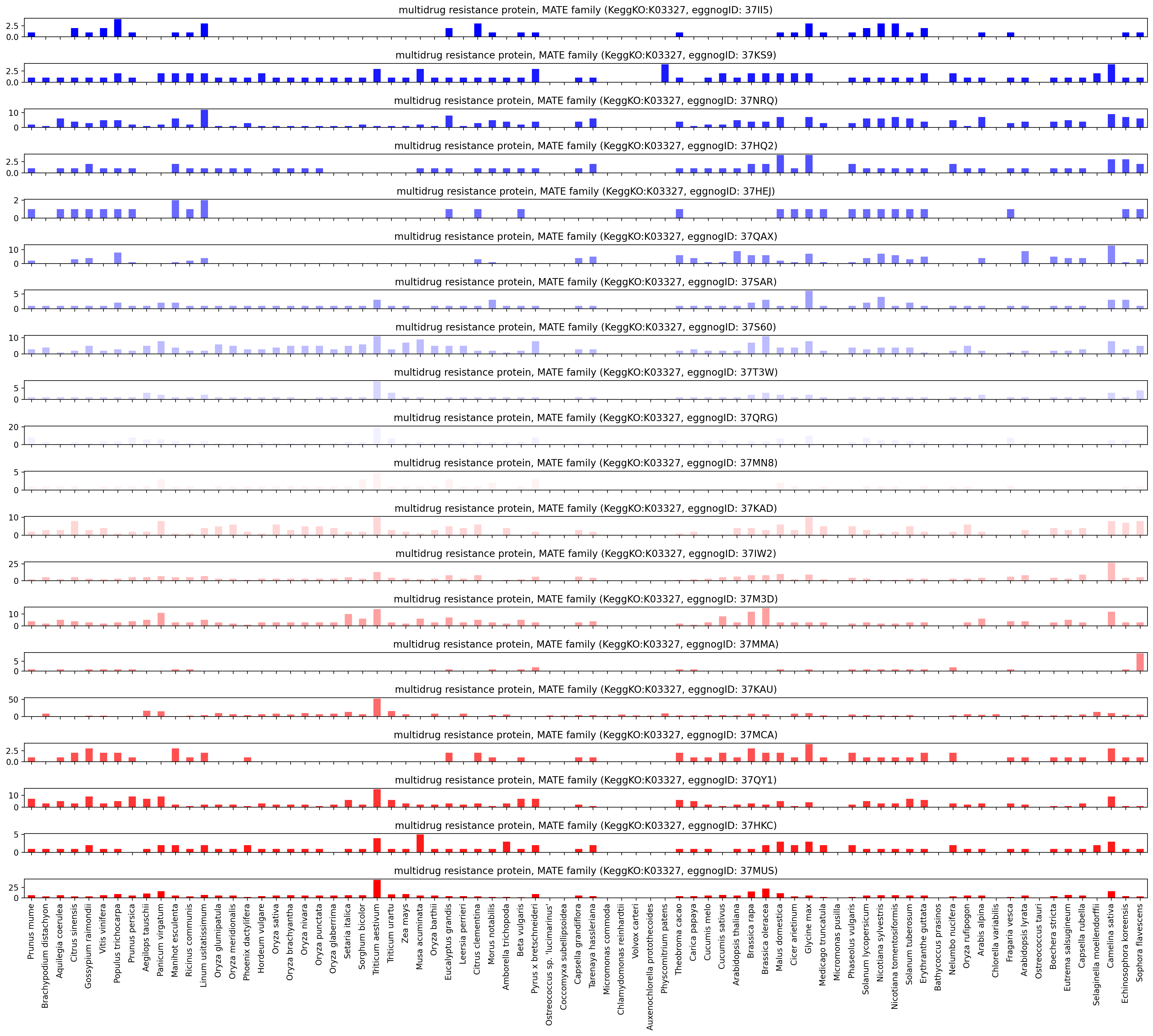
**

**Supplementary Figure 6.** Copy number plot of MATE orthologs (KEGG ID: K03327) within the gene family profile. Notably, the 37MMA species shows a remarkable specificity for *S. flavescens*.


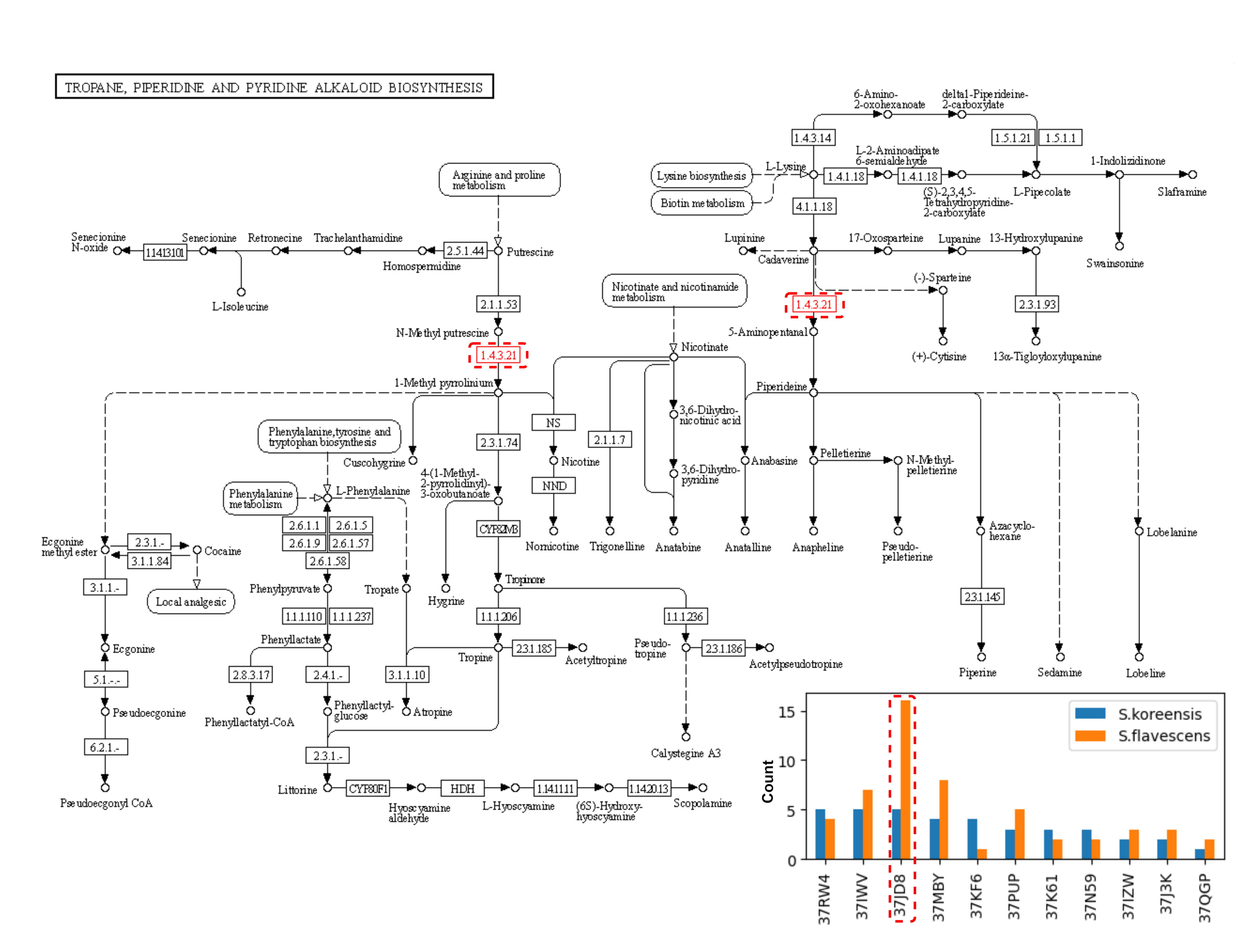


**Supplementary Figure 7**. Increased copy number of amine oxidase (eggnog: 37JD8) in *S. flavescens* compared to *S. koreensis* based on Kegg pathway [36], map00960 (Tropane, piperidine and pyridine alkaloid biosynthesis), highlighting differences in alkaloid synthesis between the two species. The red boxes indicate the amine oxidase in pathway and the bar plot of the copy numbers of gene families.
